# The use of LiF (TLD‐100) as an out‐of‐field dosimeter

**DOI:** 10.1120/jacmp.v8i4.2679

**Published:** 2007-09-24

**Authors:** Stephen F. Kry, Michael Price, David Followill, Firas Mourtada, Mohammad Salehpour

**Affiliations:** ^1^ Department of Radiation Physics The University of Texas M.D. Anderson Cancer Center Houston Texas U.S.A.

**Keywords:** TLD, out‐of‐field, secondary, neutrons, dosimeter

## Abstract

The commonly used thermoluminescent dosimeter TLD‐100 (Harshaw Chemical Company, Solon, OH) responds not only to photons and electrons, but also to neutrons that are produced during high‐energy therapies. As a result, TLD‐100 measurements outside of the treatment field are suspect when high‐energy radiation is used. Although alternatives such as TLD‐700 do not respond to neutrons, specialty dosimeters of this kind are expensive and are not routinely used in most clinics. In the current study, we examined the accuracy of TLD‐100 in measuring the out‐of‐field photon dose as a function of treatment energy.

To determine the accuracy of TLD‐100 as compared with TLD‐700, TLD‐100 was irradiated outside of the treatment field by medical accelerators operated at 6, 10, 15, and 18 MV. In an effort to eliminate the response of TLD‐100 to neutrons, TLD capsules were encased in varying thicknesses of cadmium foil (0.25 – 0.75 mm) before being irradiated at 18 MV. The out‐of‐field TLD‐100 was found to be accurate at 6 MV and 10 MV, but to be substantially over‐responsive at 15 MV and 18 MV (by up to 1063% relative to TLD‐700). By wrapping the TLD‐100 in up to 0.75 mm of cadmium, it was possible to drastically reduce (down to 39% on average) the over‐response of the TLD‐100; however, total removal of the over‐responsiveness was not possible. Although TLD‐100 is well suited for measuring out‐of‐field dose at energies as high as 10 MV, at higher energies (15 MV or greater), this dosimeter over‐responds substantially and should not be used. Although encasing the TLD in cadmium minimized over‐response to a degree, the reduction was not sufficient to make TLD‐100 viable for measuring out‐of‐field dose at high treatment energies.

PACS numbers: 87.52.‐g, 87.53.‐j, 87.53.‐Dq

## I. INTRODUCTION

Radiation therapy with medical accelerators is performed at several photon beam energies—commonly 6, 10, 15, and 18 MV. Irradiation at treatment energies at or above 10 MV is associated with neutron production, which becomes more pronounced with increasing X‐ray energy.^(1)^

Photon and electron doses delivered during radiation therapy are often measured with thermoluminescent dosimeters (TLDs). One standard form of TLD, TLD‐100 (Harshaw Chemical Company, Solon, OH),^(2)^ contains a natural abundance of ${}^{6}\text{Li}$ (7.5%) and ${}^{7}\text{Li}$ (92.5%); however, TLD‐100 responds not only to photons and electrons, but also to neutrons, particularly thermal neutrons.^(3)^ Any TLD measurements at high X‐ray energies will therefore include a response to the neutron fluence generated by the accelerator. Although the neutron dose can be resolved from a TLD‐100 signal, the procedure is not simple, and the resulting measurements have high uncertainty.^(4)^ It is desirable for a TLD to be able to measure solely the photon component of the dose.

Within the primary treatment field of a medical accelerator, the neutron dose is negligible as compared with the photon dose. The response by TLD‐100 to neutrons is therefore also negligible, and TLD‐100 is routinely used to accurately measure the photon dose within a treatment beam at any energy. However, outside the treatment field, the relative importance of neutrons increases. Thus, measurements determined by TLD‐100 of the photon dose far from the treatment field have been observed to be highly inaccurate at 18 MV.^(5)^

One alternative for measurement of the out‐of‐field photon dose is to use a specialty TLD such as TLD‐700 (Harshaw), which contains 99.99% ${}^{7}\text{Li}$ and is unresponsive to neutrons.^(^ ^3^ ^,^ ^6^ ^)^ Most radiation therapy clinics have TLD‐100 on hand, but TLD‐700 is expensive and is not routinely used or available in the clinics. Moreover, the use of TLD‐700 requires a unique characterization of energy response, linearity, and fading, which is time‐consuming, precluding its use by many clinics. This obstacle is particularly inconvenient if only a few measurements of out‐of‐field dose are required. An example would be the study of a woman who is pregnant and receiving radiotherapy, because the fetal dose is often monitored using TLD. However, if high‐energy therapy is used, TLD‐100 measurements become suspect.

An alternative to calibrating and using TLD‐700 might be to encase common TLD‐100 in cadmium foil. Cadmium has a very high absorption cross‐section for thermal neutrons, possibly allowing for isolation of the photon dose. The present study therefore had two objectives. The first was to determine the energies at which the use of TLD‐100 to accurately measure the photon dose outside the treatment field is appropriate. The second was to study the feasibility of isolating the photon dose by wrapping the TLD‐100 in cadmium foil to filter thermal neutrons.

## II. MATERIALS AND METHODS

Measurements for this study were made using Harshaw TLD‐100 and TLD‐700 irradiated by Varian accelerators (Varian Medical Systems, Palo Alta, CA) operated at 6, 10, 15, or 18 MV. For all locations where the dose was measured, 3 TLDs were irradiated, with the average signal being determined. All TLDs were read at the Radiological Physics Center [RPC (Houston, TX)], which has an established history of TLD‐100 use and analysis.^(2)^ Separate calibration of energy dependence, fading, and linearity correction was required for TLD‐700, because the RPC does not routinely use it. The correction for the energy dependence was determined by comparing the signal from a known exposure from each treatment energy to a known exposure from cobalt. The fading correction was provided by the manufacturer. The linearity correction was determined by interpolation of multiple known exposures over the range of doses received by the experimental TLDs. The uncertainty in the calibration and readings of TLD‐700 was slightly larger than that for TLD‐100. The uncertainty in the dose measured by a set of 3 TLD‐100 dosimeters was previously found to be 1.3% (1 standard deviation of the mean) when RPC procedures were followed.^(2)^ The uncertainty in the dose measured by a set of 3 TLD‐700 dosimeters was previously found to be 3% (1 standard deviation of the mean) based on the variability observed in the 3 dosimeters.^(1)^

### A. Treatment energy study

The suitability of TLD‐100 for measuring out‐of‐field photon dose at various treatment energies was investigated first. Enough TLD‐100 powder to provide 3 useful readings (approximately 60 μg total) was placed in an anthropomorphic Rando phantom (Radiology Support Devices, Long Beach, CA) at each of 10 points corresponding to various anatomic locations outside the treatment field. The distance from the central axis to the TLD ranged between 15 cm and 52.5 cm. The Rando phantom was irradiated with a complete treatment of intensity‐modulated radiation therapy for prostate [approximately 40 000 monitor units (MUs), with a field size of approximately 10×10 cm)^(1)^. The treatment plan and irradiation procedure were repeated for 6, 10, 15, and 18 MV irradiations as previously described,^(1)^ and the entire process was repeated using TLD‐700. The doses measured using TLD‐700 were taken to be the correct doses (in comparison to the doses measured using TLD‐100).

### B. Cadmium foil study

The feasibility of isolating the photon signal by wrapping capsules of TLD‐100 with cadmium foil was studied at 18 MV. Three capsules of TLD‐100 powder were placed at each of four locations in a rectangular acrylic phantom. The selected locations corresponded to distances of 17.0 cm and 51.6 cm from the central axis at depths of 3.75 cm and 11.25 cm for both distances. These four locations provided a variety of photon and neutron contributions. At 17 cm from the central axis, the photon dose is high relative to that at 51.6 cm from the central axis, and at a depth of 3.75 cm, the neutron dose is high relative to that at a depth of 11.25 cm.

Capsules of TLD‐100 were then wrapped with cadmium foil (ESPI, Ashland, OR) to achieve various thicknesses of encapsulation. The foil was 0.25 mm thick and highly malleable, making it easy to wrap around the TLD capsules. The 0.25 mm thickness was used because it is comparable to that used by foil‐activation neutron moderator systems to block thermal neutrons.^(7)^ The TLD‐100 was irradiated at the same four locations without and with multiple wrappings of cadmium foil corresponding to cadmium thicknesses of 0, 0.25, 0.5, and 0.75 mm. Capsules of TLD‐700 were also irradiated at the same locations, and the measured dose from TLD‐700 was taken to be the true value at each location.

The acrylic phantom was irradiated using 25 000 MU and a field of 10×10 cm, with the gantry at 0 degrees [International Electrotechnical Commission convention (www.iec.ch/)] to deliver measurable out‐of‐field doses to the TLDs.

## III. RESULTS AND DISCUSSION

### A. Treatment energy study

Tables 1 to 4 show the dose per MU measured with the TLD‐700 and TLD‐100 dosimeters for 6‐, 10‐, 15‐, and 18‐MV irradiations respectively. In addition, the absolute and percentage difference in dose is also shown for each measurement site and for the average of all 10 sites.

**Table 1:** Dose measurements at 6 MV

	Dose (μGy/MU)		Difference	
Distance from central axis (cm)	TLD‐700	TLD‐100	(μGy/MU)	(%)
15	19.4	19.0	−0.4	−2.1
15	17.0	18.3	1.2	7.3
17.5	13.5	13.7	0.2	1.6
25	7.6	8.2	0.6	7.5
25	8.8	8.2	−0.6	−6.3
25	8.6	8.6	0.0	0.1
27.5	5.6	6.1	0.5	8.3
35	3.7	3.7	0.0	0.7
40	2.8	3.2	0.4	13.0
52.5	2.4	2.6	0.2	7.4
Average	—	—	0.2	3.7

**Table 2:** Dose measurements at 10 MV

	Dose (μGy/MU)		Difference	
Distance from central axis (cm)	TLD‐700	TLD‐100	(μGy/MU)	(%)
15	34.5	32.0	−2.5	−7.4
15	32.6	31.3	−1.3	−4.0
17.5	26.1	25.5	−0.6	−2.2
25	11.9	12.4	0.5	4.2
25	12.2	12.2	0.0	0.1
25	11.2	11.7	0.5	4.7
27.5	10.9	10.7	−0.1	−1.1
35	6.6	6.8	0.1	1.6
40	4.7	4.9	0.3	5.9
52.5	3.0	3.4	0.4	14.5
Average	—	—	−0.3	1.6

**Table 3:** Dose measurements at 15 MV

	Dose (μGy/MU)		Difference	
Distance from central axis (cm)	TLD‐700	TLD‐100	(μGy/MU)	(%)
15	15.0	27.8	12.8	84
15	15.8	29.3	13.5	85
17.5	12.7	25.6	12.9	101
25	7.9	17.4	9.5	120
25	8.0	19.0	10.9	136
25	8.1	16.1	8.0	99
27.5	5.3	20.2	14.9	281
35	3.8	17.1	13.3	346
40	2.6	13.8	11.1	421
52.5	1.8	13.6	11.8	649
Average	—	—	11.9	233

**Table 4:** Dose measurements at 18 MV

	Dose (μGy/MU)		Difference	
Distance from central axis (cm)	TLD‐700	TLD‐100	(μGy/MU)	(%)
15	16.0	42.5	26.6	166
15	15.6	44.4	28.9	185
17.5	11.7	41.0	29.2	249
25	8.1	32.9	24.9	309
25	7.8	34.1	26.3	338
25	7.9	26.7	18.8	239
27.5	5.6	37.7	32.1	573
35	3.6	33.0	29.3	807
40	2.7	27.7	25.0	936
52.5	2.3	27.0	24.7	1064
Average	—	—	26.6	487

As expected, the dose measured at 6 MV using TLD‐100 agreed within measurement uncertainties with the dose measured using TLD‐700, because no neutron production occurs at the 6‐MV energy. Although neutrons are present at 10 MV, their contribution is small enough that measurements using TLD‐100 and TLD‐700 remain in good agreement at that energy. However, agreement was no longer the case at 15 MV, where a systematic over‐response from the TLD‐100 of approximately 12 μGy/MU was observed. Although that difference was small in absolute terms, it corresponded with a large percentage difference (from 85% to nearly 650%), because the doses were small. Agreement between the two types of TLD was even poorer at 18 MV. A systematic over‐response of almost 27 μGy/MU was observed from the TLD‐100, which corresponded to a percentage difference of between 166% and 1064%—that is, nearly 3 – 12 times the dose. Regardless of location, the TLD‐100 dosimeters all drastically over‐responded as compared with the TLD‐700 dosimeters.

It must be noted that the over‐response of the TLD‐100 does not correspond with an accurate measure of the neutron dose. The TLD‐100 over‐responded by approximately 27 μGy/MU at 18 MV. In contrast, the out‐of‐field neutron dose at that energy has previously been determined in a phantom to be in the range of 0.1 – 1 μGy/MU (assuming a radiation weighting factor of 10 for the dose equivalents determined in the literature).^(^ ^1^ ^,^ ^8^ ^,^ ^9^ ^)^ The TLD‐100 response to neutrons is not known for the system used at the RPC, nor is there any simple technique for separating the photon and neutron contributions when reading the TLD. This issue is further complicated because most of the neutron dose equivalent is delivered by fast neutrons, but most of the TLD signal originates from thermal neutrons.^(^ ^1^ ^,^ ^5^ ^)^

### B. Cadmium foil study

Table 5 shows the doses measured with TLD‐700 at 18 MV for the four locations set in the acrylic phantom for the cadmium foil study. The doses measured with TLD‐100 covered with cadmium foil are also included. Table 6 summarizes the percentage differences between the TLD‐100 and TLD‐700 readings. With no cadmium foil, the TLD‐100 readings are substantially erroneous, which is expected, considering the results of the treatment energy portion of the study (Table 4). At 51.6 cm from the central axis and at a depth of 3.75 cm (where the neutron dose is highest relative to the photon dose), the bare TLD‐100 had an 852% error relative to TLD‐700. At this location, a covering of 0.25 mm cadmium reduced the over‐response to 85%. This large percentage error corresponds to a relatively small absolute error (9 μGy/MU) because the dose is very small at that point. A covering of 0.5 mm or 0.75 mm cadmium at the same location led to a further, albeit small, increase in the accuracy of the TLD‐100 reading. The thicker cadmium (0.5 mm) reduced the error from 852% to 67% (7 μGy/MU).

**Table 5:** Effect of cadmium foil on the dose measured by TLD‐100 at 18 MV

Measurement location		Dose (μGy/MU)				
Distance^a^ (cm)	Depth^b^ (cm)	TLD‐700	TLD‐100			
			No Cd	0.25‐mm Cd	0.5‐mm Cd	0.75‐mm Cd
17	3.75	172.3	327.8	206.4	202.9	201.3
17	11.25	134.2	216.1	163.1	165.0	158.5
51.6	3.75	10.1	96.5	18.7	16.9	16.9
51.6	11.25	7.7	57.0	12.0	12.0	11.9

a Distance from the central axis.

b Depth from phantom surface.

**Table 6:** Difference between measured doses from TLD‐100 covered in various thicknesses of cadmium and TLD‐700

Measurement location		Difference compared with TLD‐700 [% (absolute^c^)]			
Distance^a^ (cm)	Depth^b^ (cm)		TLD‐100		
		No Cd	0.25‐mm Cd	0.5‐mm Cd	0.75‐mm Cd
17	3.75	90 (155)	20 (34)	18 (31)	17 (29)
17	11.25	61 (82)	22 (29)	23 (31)	18 (24)
51.6	3.75	852 (86)	85 (9)	67 (7)	66 (7)
51.6	11.25	637 (49)	55 (4)	54 (4)	54 (4)

a Distance from the central axis

b Depth from phantom surface

c Absolute difference in units of μGy/MU

With the thickest covering of cadmium (0.75 mm), the average percentage difference over all four points of measurement was 39%. Nearer the treatment field (17 cm distance), where the dose from photons dominates the contribution by neutrons, the over‐response of the TLD‐100 was less than 20% for the dosimeter covered in 0.75 mm of cadmium. Far from the treatment field, the percentage difference was nearly four times higher.

Generally, out‐of‐field dose measurements are less accurate and require less accuracy than in‐field measurements. However, the accuracy that we attained using TLD‐100 would likely be inadequate for any dose measurement, regardless of the thickness of cadmium cover. Although the average percentage error at 18 MV was reduced from 410% with no cadmium to 39% with 0.75 mm of cadmium, 39% is still a substantial error.

The over‐response observed with the cadmium‐wrapped TLD‐100 likely results from a combination of factors. Although TLD‐100 responds primarily to thermal neutrons, it will nevertheless detect some dose from fast neutrons that are not blocked by the cadmium foil. There will also be an undesired response from fast neutrons that penetrate the cadmium foil but are then thermalized in the plastic capsule that holds the TLD powder or in the TLD itself. These thermalized neutrons can then be captured by the TLD.

These effects are sufficient to make a notable contribution to the over‐response of TLD‐100.

## IV. CONCLUSIONS

We found TLD‐100 to be a suitable detector for measuring out‐of‐field dose for treatment energies up to and including 10 MV. At higher energies, TLD‐100 severely over‐responds and does not produce a useful measure of the dose. Thus, at the higher energies, TLD‐100 should not be used outside of the treatment field.

An attempt was made to block the thermal neutrons produced during high‐energy therapies by encasing the TLD in cadmium foil. Although the wrapping drastically reduced the over‐response of the TLD‐100, the reduction was insufficient for TLD‐100 to be clinically viable as a dosimeter for out‐of‐field dose measurements in those circumstances. An alternative detector must be used outside of the treatment field at high treatment energies.

## ACKNOWLEDGMENTS

The authors thank Gary Ezzell, PhD, Arthur Boyer, PhD, and the faculty and staff at the Mayo Clinic (Scottsdale, AZ) and the Stanford Clinic (Stanford, CA), where some measurements were conducted.

This work was supported by the Rosalie B. Hite Foundation.
